# Endoplasmic Reticulum Stress in Systemic Lupus Erythematosus and Lupus Nephritis: Potential Therapeutic Target

**DOI:** 10.1155/2023/7625817

**Published:** 2023-08-31

**Authors:** Hui-Yuan Li, Li-Feng Huang, Xiao-Rong Huang, Dan Wu, Xiao-Cui Chen, Ji-Xin Tang, Ning An, Hua-Feng Liu, Chen Yang

**Affiliations:** Guangdong Provincial Key Laboratory of Autophagy and Major Chronic Non-communicable Diseases, Key Laboratory of Prevention and Management of Chronic Kidney Disease of Zhanjiang City, Institute of Nephrology, Affiliated Hospital of Guangdong Medical University, Zhanjiang, Guangdong 524001, China

## Abstract

Systemic lupus erythematosus (SLE) is a complex autoimmune disease. Approximately one-third to two-thirds of the patients with SLE progress to lupus nephritis (LN). The pathogenesis of SLE and LN has not yet been fully elucidated, and effective treatment for both conditions is lacking. The endoplasmic reticulum (ER) is the largest intracellular organelle and is a site of protein synthesis, lipid metabolism, and calcium storage. Under stress, the function of ER is disrupted, and the accumulation of unfolded or misfolded proteins occurs in ER, resulting in an ER stress (ERS) response. ERS is involved in the dysfunction of B cells, macrophages, T cells, dendritic cells, neutrophils, and other immune cells, causing immune system disorders, such as SLE. In addition, ERS is also involved in renal resident cell injury and contributes to the progression of LN. The molecular chaperones, autophagy, and proteasome degradation pathways inhibit ERS and restore ER homeostasis to improve the dysfunction of immune cells and renal resident cell injury. This may be a therapeutic strategy for SLE and LN. In this review, we summarize advances in this field.

## 1. Introduction

Systemic lupus erythematosus (SLE) [1, 2] is an autoimmune disease associated with multiple factors, including genetic, environmental, and lifestyle factors. Multiple systems and organs are involved in SLE and are driven by abnormal innate immunity and adaptive immunity. The accumulation of autoantibody deposition in the kidney leads to renal inflammation and the destruction of renal structure and function, which is termed lupus nephritis (LN) and is the main cause of morbidity and mortality in SLE patients [3].

More than 1 million patients with SLE exist in China, which ranks as the country with the highest number of SLE patients in the world. Compared with European and American populations, the incidence of SLE in the Chinese population is higher, the onset age is earlier, and the disease is more severe [4, 5]. Currently, the pathogenesis of SLE and LN remains unclear, although a large number of studies have shown that it may be related to genetic, environmental, and drug factors, lymphocyte abnormalities, abnormal complement activation, and autoantibody production [6, 7]. The treatment of SLE is extremely challenging with slow progress, because of the difficulty of early and precise prevention, and the use of therapies that mainly rely on hormone and immunosuppressive strategies [8–10]. Therefore, it is imperative to identify the pathogenic mechanisms and explore new therapies for SLE and LN.

The endoplasmic reticulum (ER) is the largest intracellular organelle and is a site for protein synthesis, lipid metabolism, and calcium storage. Disruption of ER homeostasis activates the unfolded protein response (UPR), in which unfolded or misfolded proteins accumulate in the ER, resulting in an ER stress (ERS) response [11]. ERS is involved in the pathogenesis of SLE and LN via the activation of immune cells through multiple inflammatory signaling pathways and mediating the injury of renal resident cells [12, 13] This review aims to summarize the recent progress in understanding the role of ERS in the occurrence and development of SLE and LN and emphasize the potential of targeting ER as a therapeutic strategy for SLE and LN.

## 2. Overview of ERS

The ER is the largest organelle in eukaryotic cells. It is composed of interconnected tubular and lamellar lumens that form a network of interconnected ducts [14, 15]. The ER is a highly dynamic organelle that is a site for the synthesis, folding, modification, packaging, transport, and integration of various molecules that contribute to stress responses, such as proteins, lipids, phospholipids, cholesterol, and oligosaccharides. It is also a reservoir of calcium ions [14, 16].

When stimulated by the endogenous or exogenous factors, such as ischemia and hypoxia, infection, drug toxicity, and calcium imbalance, unfolded or misfolded proteins in the ER rapidly accumulate, which activates the UPR. The ERS response is induced to maintain the ER balance [17–19]. In the physiological state, three classical ERS pathway sensors on the ER membrane, inositol-requiring enzyme-1 (IRE1), protein kinase R- (PKR-) like ER kinase (PERK), and activated transcription factor 6 (ATF6), bind to glucose-regulated protein 78 (GRP78, also known as BiP), which inactivates the downstream signaling pathways. When ERS occurs, GRP78 dissociates from the ERS sensors IRE1, ATF6, and PERK, and binds to unfolded proteins that then activate IRE1, PERK, and ATF6 signaling pathways via different downstream target genes to enhance the correct folding of proteins, to promote the degradation of misfolded proteins, and reduce the cell damage [20, 21]. However, persistent ERS initiates apoptosis to eliminate the damaged cells [22].

In addition to the UPR, other ER quality control (ERQC) systems are present inside the cell, such as the ER-associated protein degradation (ERAD) and autophagy–lysosome pathway [11, 16]. The ER does not contain a degradation apparatus; therefore, most misfolded proteins produced by ERS are mislocated on the membrane and degraded by the cytosolic 26S proteasome via the ERAD pathway or by the autophagy–lysosome pathway. Some misfolded proteins are also transported to lysosomes for clearance by the ER-to-lysosomal-associated degradation pathway [23, 24].

### 2.1. IRE1*α* Pathway

The IRE1*α* pathway is the most conserved in the UPR, with dual enzyme activity involving IRE1*α* and IRE1*β* present in the mammals [21]. When ERS occurs, IRE1 oligomerization induces the kinase domain that is autophosphorylated to form a dimer, which catalyzes the cleavage and activation of the X-box binding protein 1 (XBP1) mRNA into XBP1s. After entering the nucleus, the transcription of stress proteins in the ER lumen and ERAD is upregulated, and finally ERS is alleviated [25]. In addition, Phospho-IRE1*α* (P-IRE1*α*) can degrade the ribosome-related mRNA by dependent decay (RIDD), which hinders the transcription and translation of unfolded proteins and reduces the ERS. However, during sustained ERS, P-IRE1*α* can recruit tumor necrosis factor receptor-associated adaptor protein 2 (TRAF2), which promotes inflammation and apoptosis by phosphorylating the c-Jun N-terminal kinase (JNK) pathway and activating the nuclear factor-*κ*B (NF-*κ*B) signaling pathway [25, 26].

### 2.2. PERK Pathways

The mode of activation of the PERK pathway is similar to that of IRE1*α*. The N-terminus of the PERK protein can sense ERS signals, and the C-terminus has filament/threonine protein kinase domain, but no endonuclease activity. When ERS occurs, PERK dissociates from GRP78 and can phosphorylate eukaryotic initiation factor 2*α* (elF2*α*) to inhibit cyclin D1 translation specifically, thereby inhibiting protein synthesis and reducing protein accumulation in the ER [19]. Moreover, phosphorylated elF2A can enhance the translation of ATF4, GRP78, and other mRNAs to promote protein folding in the ER to restore ER homeostasis [27]. When ERS persists, the PERK pathway upregulates the expression of the C/EBP homologous protein gene (CHOP) and promotes P-EIF2*α* dephosphorylation, and inhibits its reverse transcription, resulting in the induction of apoptosis-related genes encoding BCL2-interacting mediator of cell death and p53-upregulated modulator of apoptosis [28–30].

### 2.3. ATF6 Pathways

ATF6 is a DNA transcriptional activation protein that contains a basic zinc-finger domain (BZIP). The protein localizes to the ER membrane. There are two ATF6 subtypes in mammals, ATF6*α* and ATF6*β*, with multiple GRP78-binding sites and two Golgi localization signals, which can sense stress signals [25]. When ERS occurs, ATF6*α* dissociates from GRP78 and is transferred to the Golgi apparatus via vesicle trafficking. It is cleaved by Site-1 and Site-2 proteases in the Golgi apparatus, which releases the BZIP domain that then migrates to the nucleus, where it binds to ATF/CRE and ERS response elements. ER chaperone gene transcription, ERAD protein translation, and lipid biosynthesis are then induced to alleviate ERS [26, 31].

## 3. ERS in Immune Cells in SLE and LN

**Figure 1:**
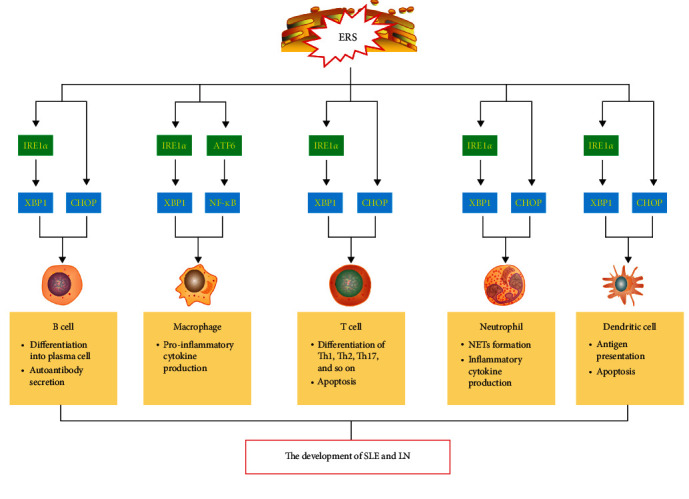
A schematic diagram of ERS in immune cells in SLE and LN. An abnormal ER stress (ERS) pathway is closely related to the autoimmune diseases, such as SLE and LN. ERS promotes B cells differentiate into plasma cell and increased antibody secretion by activating IRE1–XBP1 and CHOP pathways. TLR promotes the production of proinflammatory cytokines in macrophages by activating the IRE1*α*XBP1 pathway. ERS can also activate the ATF6 pathway to further activate the NF-*κ*B inflammatory pathway and promote the secretion of inflammatory factors in macrophages. ERS drives T cell differentiation by activating the IRE1XBP1 pathway and can also activate the CHOP pathway to promote the T cell apoptosis. ERS participates in the formation and release of neutrophil extracellular traps (NETs) and the production of inflammatory cytokines in neutrophils. ERS participates in antigen presentation and cytokine secretion of DCs by activating the IRE1XBP1 and CHOP pathways.

ERS plays an important role in the immune system. An abnormal ERS pathway is closely related to autoimmune diseases [32]. A large number of autoantibodies produced in SLE can aggravate the ER burden and activate the ERS [33, 34]. Studies have found that ERS affects the survival, activation, differentiation, and effector functions of immune cells, including B cells, macrophages, T cells, dendritic cells, and neutrophils, resulting in a dysregulation of immune homeostasis and generating autoimmune diseases [35–37]. Clinical studies have also found that ERS-related proteins IRE1, PERK, and CHOP are downregulated in SLE patients, and XBP1 and midbrain stellate cell-derived neurotrophic factor (MANF) are upregulated, suggesting that these proteins may be involved in the pathogenesis of SLE [38]. ERS in immune cells in SLE and LN is summarized in Figure 1.

### 3.1. ERS in B Cells

Many studies have found that the pathogenesis of SLE is closely related to the B cell abnormalities and the production of autoantibodies [39]. Antibodies that target the ERS-related protein GRP78 can be detected in the blood of SLE patients, and the expression of GRP78 and XBP1 is increased in plasma cells that secret antibodies, suggesting that the ERS may promote the differentiation of B cells into plasma cells to increase the secretion of antibodies [40, 41]. It has also been reported that activation of the IRE1–XBP1 pathway is required for ER expansion and antibody secretion by the plasma cells [42, 43]. Gene expression profile analysis have shown that XBP1-deficient B cells could not upregulate most of the genes encoding the secreted antibodies. Knockdown of XBP1 in mice significantly inhibited plasma cell differentiation and decreased IgM synthesis and antibody secretion [44, 45]. In XBP1 and IRE1 knockout mice, the secretion of immunoglobulin IgG by plasma cells was significantly reduced [46].

Interestingly, although ATF6 was activated in B cells stimulated by lipopolysaccharide (LPS), ATF6 deficiency does not affect antibody secretion in B cells in contrast to the IRE1XBP1 pathway [47]. Similarly, PERK knockdown in mice had no considerable effect on B cell differentiation and antibody secretion [30, 48]. ERS apoptosis-related genes such as CHOP, caspase-4, calmodulin calponin, and Calp were markedly increased in patients with SLE [49, 50]. The expression of CHOP increases during the early differentiation of B cells and IgM is produced in the apoptotic response mediated by the CHOP. IgM secretion was also significantly reduced after CHOP knockdown in B cells [51, 52]. These findings indicate that CHOP not only participates in the differentiation of B cells into plasma cells but also promotes the antibody secretion of the B cells.

### 3.2. ERS in Macrophages

The relationship between SLE pathogenesis and macrophages is mainly characterized by multiple cell phenotypes and dysfunction [53]. A number of recent studies have found that polarization imbalance and abnormal activation of M1/M2 macrophages are closely related to the occurrence and development of SLE. Immune complex forming microparticles (MP-IC) [54], extracellular vesicles [54], and high-mobility group protein B1 (HMGB1) [55] were found to contribute to the polarization of M1 macrophages, which play a pro-inflammatory role in the pathogenesis and severity of SLE [56]. When the balance between M1/M2 phenotypes was restored, SLE disease activity was improved, resulting from the elevated anti-inflammatory activity of macrophages [56].

It has been reported that the toll-like receptor (TLR) signaling pathway in macrophages can activate the ERS response. XBP1s is a positive regulator of TLR responses in macrophages. TLR2 and TLR4 can activate the IRE1*α–*XBP1s pathway to produce the pro-inflammatory cytokines, such as interleukin-6 (IL-6), tumor necrosis factor (TNF), and interferon-*β* (IFN-*β*) [57, 58]. The specific knockdown of XBP1 in macrophages resulted in decreased production of IL-6, TNF, and IFN-*β*, while in contrast, overexpression of XBP1 resulted in increased IFN-*β* production [18, 59]. In addition, the activation of IRE1*α*-dependent glycogen synthase kinase 3*β* (GSK3*β*) via the IRE1*α–*XBP1 pathway is related to the production of IL-1*β*. GSK3*β* can inhibit the cleavage of XBP1 and the transcription of TNF, thereby attenuating the ERS-mediated inflammatory response [60]. In vitro, the downregulation of ATF6 in macrophage inhibited NF-*κ*B activity and, consequently, reduced the TNF-*α* and IL-6 production in these cells [18, 61]. These findings suggested that the ERS response may promote the differentiation of macrophages into the M1 type, activate the NF-*κ*B inflammatory pathway, and increase the secretion of inflammatory factors involved in the progression of SLE through TLR signaling.

### 3.3. ERS in T Cells

In recent years, it has been found that during ERS, autoimmune cells can induce the immune responses by recognizing unfolded or misfolded proteins as antigens, which can promote the development of autoimmune diseases [62, 63]. T lymphocytes are the main effector cells in cellular immunity and produce cytokines to mediate inflammatory responses by changing their function and phenotype. However, a little is known about the driving force of behind T-cell differentiation within this plastic spectrum [64]. Franco et al. [65] and Kemp and Poe [66] showed that the ERS response includes antigen recognition during initial T cell differentiation, which may be the key event driving the plastic differentiation of T cells. ERS can activate primary CD4+ T cells and cause them differentiate into Th1, Th2, Th9, Th17, Th22, Tfh, and Treg cells, as well as other cell subsets by promoting major histocompatibility complex (MHC) Class II antigen-presenting cells (APCs). Lineage-specific cytokines are produced to change the functional phenotypes of these immune cells [67, 68].

Clinical studies have shown an abnormal UPR in T cells in patients with SLE, suggesting that T lymphocytopenia may be related to ERS, which can regulate T cell differentiation through metabolic pathways [69–71]. Increased expression of CHOP and decreased expression of GRP78 may contribute to the apoptosis of T cells in patients with SLE [69]. Other studies have found that the ERS-related protein XBP1 is essential for T cell differentiation and plays a key role in the differentiation of Th17 cells and CD8+T cells [18, 72]. ERS inhibitors (such as 4-phenylbutyric acid, 4-PBA) can significantly reduce the levels of anti-dsDNA antibodies and serum TNF-*α*, which delays the progression of SLE [73, 74]. ERS and autophagy-coupling pathways mediate Th17 activation promoted by peptididylarginine deiminase type 2 [75].

### 3.4. ERS in Neutrophils

In addition to their antimicrobial function, neutrophil extracellular traps (NETs) are involved in the progression of autoimmune diseases through the activation and differentiation of macrophages, dendritic cells, and T cells [76, 77]. Studies have found that NET formation is closely related to the SLE pathogenesis [78, 79]. The ribonucleoprotein immune complex (RNP-IC) found in SLE induces NET formation by promoting the mitochondrial reactive oxygen species (ROS) production [80, 81]. Low-density granulocytes (LDGs) are a subpopulation of pro-inflammatory neutrophils found in patients with SLE and are involved in the pathogenesis of lupus by disrupting endothelial cells and increasing the production of pro-inflammatory cytokines and type I interferon [82].

ERS has been reported to be involved in the NET formation and release in neutrophils [83]. In the neutrophils isolated from the blood of patients with SLE, increased activity of IRE1*α* was detected. In multiple SLE mouse models, inhibition of IRE1*α* reduced NET release and delayed disease progression [33]. In addition, studies have shown that IRE1*α* activates neutrophil antimicrobial activity, including the production of IL-1*β* and the formation of NETs through increased production of ROS and activation of caspase-2 [84]. Tumurkhuu et al. [85] showed that inflammatory markers were significantly upregulated in neutrophils isolated from patients with SLE with diffuse alveolar hemorrhage (DAH) and that ERS-related genes were highly expressed in alveolar epithelial cells. Additionally, coculture of human neutrophils and a human lung epithelial cell line (BEAS-2B) showed that the neutrophils from patients with SLE significantly upregulated the ERS-related indicators in epithelial cells as compared to the neutrophils from healthy controls, suggesting that NETs play an important role in SLE complicated with DAH by inducing an ERS response [85].

### 3.5. ERS in Dendritic Cells

Dendritic cells (DCs) are the most functional APCs in humans. General antigen presentation can be divided into several stages: adhesion, antigen-specific activation, costimulation, cytokine production, and signal transduction [86]. DCs are equivalent to messengers that transmit antigen information to activate T cells [87, 88]. DCs internalize protein antigens as peptides that enter the ER and bind to histocompatibility type I (MHC-I) protein complexes, which are then transported to the cell surface for cross-presentation [89]. The UPR sensor IRE1 is a key regulator of APC homeostasis, and XBP1 plays an important role in DCs development and survival [90]. Studies have shown that the IRE1*α–*XBP1 pathway is continuously activated in infiltrating DC under ERS conditions, which can deplete MHC-I heavy chain mRNA and reduce antigen cross-presentation through regulated IRE1-dependent decay [90, 91]. Chaudhary et al. [92] showed that the IRE1*α*-XBP1 branch of the UPR inhibits IFN-*α* production by TLR7- or TLR9-activated plasmacytoid DCs. In addition, XBP1 promotes triglyceride biosynthesis in DCs, leading to abnormal lipid accumulation and impaired antigen presentation [93]. Under ERS conditions, TLR agonists increase the expression of IL-23 in DCs by enhancing the binding of CHOP to the IL-23 promoter, whereas downregulation of CHOP decreases the expression of IL-23 [94].

Renal dendritic cells mainly function as powerful APCs and regulate the inflammation [95]. DCs infiltrate into the kidneys where they form tertiary lymphoid structures to amplify inflammation [96]. Studies have shown that hyperactive B cells and plasmacytoid DCs produce IFN-*α* in LN [97, 98]. During SLE pathogenesis, an “automatic” regulatory feedback mechanism between pDCs and regulatory B (Breg) cells is characteristic. pDCs release IFN-*α* and CD40 to promote the B-cell differentiation and IL-10 production, and Breg cells, in turn, inhibit the pDC production of IFN-*α* by releasing IL-10 [99, 100]. These results suggest that ERS is related to DC function, possibly through antigen presentation and cytokine secretion.

## 4. ERS in Renal Resident Cells in LN

**Figure 2:**
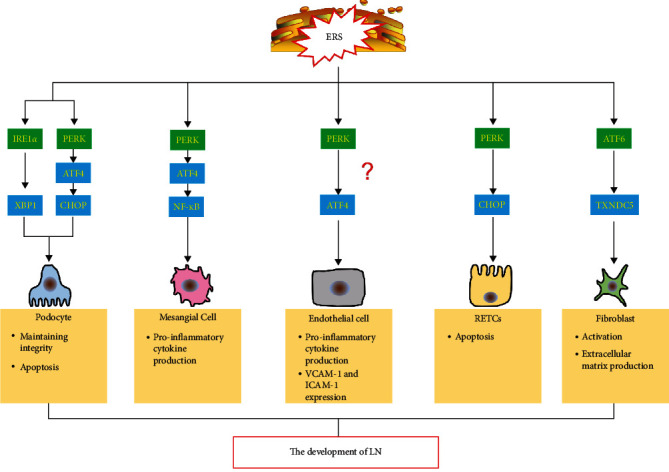
A schematic diagram of ERS in renal resident cells in LN. ER stress (ERS) is also involved in damage of renal resident cells in LN. The IRE1–XBP1 pathway plays an important protective role in maintaining the integrity of podocyte. ERS can activate the PERK–ATF4CHOP pathway to induce the podocytes apoptosis. ERS can induce the NF-*κ*B inflammatory pathway by activating the PERK–ATF4CHOP pathway and promote the secretion of inflammatory factors in mesangial cells. ERS may promotes the expression of intercellular adhesion molecule-1 (ICAM-1) and vascular cell adhesion molecule-1 (VCAM-1) and cytokine production in glomerular endothelial cells by activating the PERKATF4 pathway. ERS promotes apoptosis of renal tubular epithelial cells (RTECs) by activating the PERK–CHOP signaling pathway. ERS promotes the production of a large amount of extracellular matrix (ECM) by fibroblasts through the activation of the ATF6TXNDC5 signaling pathway.

ERS not only participates in immune disorders in SLE but is also involved in damage of renal resident cells. The critical role of ERS in acute kidney injury and chronic kidney diseases were well-reviewed in a recent study [101]. However, at present, a large gap exists in our knowledge of the role of ERS in renal resident cells in LN. Below, we summarize what is known on this topic (Figure 2).

### 4.1. ERS in Podocytes

Podocytes are highly differentiated epithelial cells that constitute an important component of the glomerular filtration barrier. The fusion and disappearance of foot processes, apoptosis, and shedding of podocytes may lead to proteinuria [102–104]. A recent study has shown that activation of IRE1*α* has a cytoprotective effect against podocyte injury in an adriamycin-induced nephropathy model [105]. These results suggested that the IRE1–XBP1 pathway plays a cytoprotective role in maintaining podocyte integrity [105]. In contrast, activation of the PERK–ATF4CHOP [106] and PERKEIF2*α* [107] signaling pathway induces podocyte apoptosis [101]. Specific knockdown of Xbp1 and Sec63 can induce activation of the JNK pathway, leading to podocyte apoptosis, the disappearance of foot processes, reduction of podocyte number, and induction progressive albuminuria [108, 109]. Additionally, podocyte cyclooxygenase-2 (COX-2) participates in the ATF4 pathway during ERS in LN, while downregulation of ATF4 inhibits the LN-induced COX-2 overexpression. These results suggest that inhibition of the ATF4 pathway during ERS may be a potential therapeutic target for LN treatment [110].

### 4.2. ERS in Mesangial Cells

Mesangial cells make up approximately 30%–40% of the total cells in the glomeruli. Mesangial cells along with the mesangial matrix form the glomerular basement membrane (GBM), whose primary function is filtration [111]. ERS plays a key role in the inflammatory response of human mesangial cells that is induced by anti-dsDNA antibodies and participates in the inflammatory response and fibrosis process involved in LN [112–114], although few studies have been conducted on this topic. Anti-dsDNA antibodies can significantly upregulate the expression of the ERS proteins GRP78, P-PERK, P-EIF2*α*, and ATF4 in mesangial cells, resulting in enhanced expression of the pro-inflammatory mediators IL-1*β*, TNF-*α*, and monocyte chemotactic protein-1 via activation NF-*κ*B, TLR4, and JAK signaling pathways. Treatment of mesangial cells with ERS inhibitors can downregulate the expression of inflammatory factors and alleviate the progression of SLE [12, 114].

### 4.3. ERS in Glomerular Endothelial Cells

Glomerular endothelial cells (ECs) are innate cells of the glomeruli that regulate the glomerular filtration. Studies have demonstrated that EC activation and dysfunction play important roles in the development of LN [115]. In experimental LN models, ECs are activated and releases inflammatory mediators [116]. These inflammatory mediators can promote the upregulation of adhesion molecules, such as intercellular adhesion molecule-1 and vascular cell adhesion molecule-1, and promote leukocyte adhesion and migration in glomeruli, leading to glomerular inflammation and glomerulosclerosis [117, 118]. Few studies have examined the relationship between ERS and endothelial dysfunction in the LN [119]. Russell et al. [120] and Oates et al. [121] found that human glomerular endothelial cells in SLE-induced neutrophil chemotaxis and adhesion and further aggravated glomerular lesions through ERS and oxidative stress pathways.

### 4.4. ERS in Renal Tubular Epithelial Cells (RTECs)

Renal tubular epithelial cells (RTECs) are resident cells in the tubulointerstitium of the kidneys that have plastic morphology and function [122]. In response to anti-dsDNA antibodies, RTECs can transform into mesenchymal cells and produce pro-inflammatory cytokines and chemokines to regulate renal tubulointerstitial immune cell responses [123]. Currently, few studies have investigated the role of ERS and RTECs in SLE pathogenesis [124, 125]. It has been reported that ERS is involved in proteinuria-induced apoptosis in RTECs. Proteinuria upregulates the expression of GRP78 and CHOP in the PTEC. In addition, persistent ERS induced by albumin overload can lead to the transformation of RTECs into mesenchymal cells via activation of the PERK–CHOP signaling pathway, which contributes to the renal fibrosis [126]. Wu et al. [127] found that albumin significantly upregulated the expression of GRP78 in mouse RTECs and induced apoptosis in these cells by calpain-mediated caspase-12 activation.

### 4.5. ERS in Fibroblasts

Studies have highlighted the functional heterogeneity and plasticity of renal resident fibroblasts, as well as their important role in the progression of kidney diseases [128, 129]. However, no recent study has reported the role of ERS in renal resident fibroblasts in LN. In response to stress, renal resident fibroblasts transdifferentiate into myofibroblasts, express *α*-smooth muscle actin (*α*-SMA), and produce a large amount of extracellular matrix, which can lead to renal fibrosis. Renal resident fibroblasts also can produce pro-inflammatory cytokines and chemokines and promote inflammatory response through activation of the NF-*κ*B pathway [130–132]. Chen et al. [133] recently identified the ER-resident protein, thioredoxin domain 5 (TXNDC5), a protein that is transcriptionally controlled by the ATF6-dependent ERS pathway and that enhances transforming growth factor-*β* (TGF-*β*) signaling activity through upregulation of the type I TGF-*β* receptor in renal fibroblasts and mediates its profibrotic effect.

## 5. Targeting ERS in the Treatment of SLE and LN

As mentioned above, ERS acts as a key part in the pathogenesis of SLE and LN. Therefore, targeting ERS may bring about a breakthrough to combat SLE and LN. The strategy targeting ERS includes improvement of protein folding with chemical chaperones, increasing degradation of misfolded proteins, and inhibiting IRE1, PERK, and ATF6 pathways [101].

### 5.1. Chemical Chaperones

4-PBA is a low-molecular weight chemical chaperone that can increase the protein folding capacity of the ER and prevent the accumulation of misfolded proteins, thereby alleviating ERS [38, 134]. 4-PBA improved splenomegaly and reduced serum anti-dsDNA antibody and inflammatory cytokine levels in lupus-proven mice. In particular, the levels of albuminuria and blood urea nitrogen, renal inflammatory cell infiltration, and immune complex deposition were significantly reduced in the mice with LN that were treated with 4-PBA [73, 135]. In addition, studies have also shown that 4-PBA can inhibit the release of the pro-inflammatory factors IL-1*β*, TNF-*α*, and IL-6 by inhibiting activation of the NF-*κ*B pathway, which alleviates the progression of SLE [136, 137].

Sodium taurodeoxycholate (TUDCA), a taurine-conjugated product of ursodeoxycholate, is a binding bile acid found naturally in bear bile, which has hepatoprotective, gallbladder-promoting, and litholytic effects, and is clinically used in the treatment of hepatobiliary diseases. In recent years, TUDCA was shown to have potential medicinal value in nonhepatobiliary diseases by inhibiting ERS [114, 138]. In diabetic nephropathy (DN), TUDCA, and 4-PBA inhibit podocyte apoptosis in vivo and in vitro by inhibiting caspase-3 and caspase-12 activation, thereby alleviating the DN progression [139, 140]. Other studies have shown that TGF-*β*1 is a key driver of renal fibrosis and is closely related to the activation of the ERS-related renal fibrosis pathway. TUDCA significantly downregulated the levels of GRP78, CHOP, *α*-SMA, and fibronectin in renal mesangial cells when subjected to TGF-*β*1. These results suggested that TUDCA inhibits ERS and alleviates the profibrotic effect of renal mesangial cells [114, 141]. However, no studies to date have reported inhibition of ERS by TUDCA for the treatment of SLE; this needs to be proven in future.

### 5.2. Inhibitors Targeting IRE1, PERK, and ATF6

Some selective regulators have been developed to target IRE1, and PERK and ATF6 pathways to alleviate ERS [32]. BI09 inhibits the ability of IRE1*α* to splice XBP1 mRNA for production of the activated transcription factor XBP1. Transient BI09 treatment prevented B cell differentiation into plasma cells, autoantibody production, autoantibody-mediated renal lesions, and proteinuria [142]. Administration of IRE1*α* inhibitor 4*µ*8C suppressed mitochondrial ROS generated in peripheral neutrophils, resulting in a reduction of plasma cell expansion and autoantibody production in a lupus-proven mouse model [33]. Similarly, targeting IRE1*α* by STF083010 protects protected against the progression of SLE and LN by preventing the B cell hyperactivity [143]. Guanabenz promoted the phosphorylation of eIF2*α* by inhibiting eIF2*α* phosphatases to enhance the PERK signaling pathway. Interestingly, guanabenz protected mice from CpG oligonucleotide-dependent cytokine shock and alleviated autoimmune symptom severity in a mouse model of pristane-induced lupus [34]. Ceapin-A7, as an ATF6*α* inhibitor, alleviates alleviated collagen-induced arthritis and bone erosion in a mouse model by suppressing the inflammatory cytokine production [144]. However, the potential applications of ATF6*α* inhibitors in SLE and LN have yet to be revealed.

### 5.3. Degradation of Accumulated Misfolded Proteins

In addition, ER proteins can be selectively degraded by autophagy and proteasomes to remove proteins and molecular chaperones that accumulate in the lumen of the ER to maintain ER homeostasis [15]. ER expansion and ERS-related proteins were upregulated when the Atg7 autophagy gene was knocked down in T lymphocytes [145] or the Atg5 autophagy gene was knocked down in B lymphocytes [146], which suggested that autophagy plays a crucial role in ER homeostasis. Other studies have shown that ERS mediated by various noncoding RNAs plays an important role in maintaining ER homeostasis [147–149]. The antioxidant melatonin is known to scavenge free radicals and increase the activity of antioxidant enzymes in vivo, suggesting melatonin is involved in the ER homeostasis and has a potential protective effect against ERS [150]. The research advances in these areas may hold promise for the treatment of LN by inhibiting ERS.

Finally, a small number of modulators of ER stress have been used in preclinical studies, including sevoflurane (NCT03561831), TUDCA (NCT02218619, NCT00771901, and NCT01877551), and 4-PBA (NCT00771901). However, none of them have been applied in rescuing SLE and LN, or any other autoimmune disease.

## 6. Conclusion and Perspective

In summary, ERS is a significant factor in the pathogenesis of SLE and LN, whereas the detailed mechanisms involved require further elucidation. Many basic research studies of ERS have been performed in animal models or in vitro in cell culture, but whether these findings can translate to humans needs further investigation. Second, distinct UPR pathways may play different roles in immune cells and renal resident cells in LN. Third, the application of other strategies for restoration of ER homeostasis, such as ERAD and the autophagy–lysosome pathway, in SLE and LN have not been investigated to date. Nevertheless, pharmacological agents targeting ERS may represent a therapeutic approach for SLE and LN, and these approaches could be used to test their effects in the clinical trials.
